# Multi‐scale network regression for brain‐phenotype associations

**DOI:** 10.1002/hbm.24982

**Published:** 2020-03-26

**Authors:** Cedric Huchuan Xia, Zongming Ma, Zaixu Cui, Danilo Bzdok, Bertrand Thirion, Danielle S. Bassett, Theodore D. Satterthwaite, Russell T. Shinohara, Daniela M. Witten

**Affiliations:** ^1^ Department of Psychiatry Perelman School of Medicine, University of Pennsylvania Philadelphia Pennsylvania USA; ^2^ Department of Statistics The Wharton School, University of Pennsylvania Philadelphia Pennsylvania USA; ^3^ Department of Psychiatry, Psychopathology and Psychosomatics RWTH Aachen University Aachen Germany; ^4^ JARA‐BRAIN, Jülich‐Aachen Research Alliance Jülich Germany; ^5^ Université Paris‐Saclay, CEA, Inria Gif‐sur‐Yvette France; ^6^ Department of Bioengineering McGill University Montreal Canada; ^7^ Department of Bioengineering School of Engineering and Applied Science, University of Pennsylvania Philadelphia Pennsylvania USA; ^8^ Department of Electrical and Systems Engineering School of Engineering and Applied Science, University of Pennsylvania Philadelphia Pennsylvania USA; ^9^ Department of Neurology Perelman School of Medicine, University of Pennsylvania Philadelphia Pennsylvania USA; ^10^ Department of Physics and Astronomy School of Arts and Science, University of Pennsylvania Philadelphia Pennsylvania USA; ^11^ Santa Fe Institute Santa Fe New Mexico USA; ^12^ Penn Statistics and Visualization Center, Department of Biostatistics, Epidemiology, and Informatics Perelman School of Medicine, University of Pennsylvania Philadelphia Pennsylvania USA; ^13^ Center for Biomedical Imaging Computing and Analytics Perelman School of Medicine, University of Pennsylvania Philadelphia Pennsylvania USA; ^14^ Department of Statistics College of Arts and Science, University of Washington Seattle Washington USA; ^15^ Department of Biostatistics School of Public Health, University of Washington Seattle Washington USA

**Keywords:** functional connectivity, multivariate analysis, network neuroscience

## Abstract

Brain networks are increasingly characterized at different scales, including summary statistics, community connectivity, and individual edges. While research relating brain networks to behavioral measurements has yielded many insights into brain‐phenotype relationships, common analytical approaches only consider network information at a single scale. Here, we designed, implemented, and deployed Multi‐Scale Network Regression (MSNR), a penalized multivariate approach for modeling brain networks that explicitly respects both edge‐ and community‐level information by assuming a low rank and sparse structure, both encouraging less complex and more interpretable modeling. Capitalizing on a large neuroimaging cohort (*n* = 1, 051), we demonstrate that MSNR recapitulates interpretable and statistically significant connectivity patterns associated with brain development, sex differences, and motion‐related artifacts. Compared to single‐scale methods, MSNR achieves a balance between prediction performance and model complexity, with improved interpretability. Together, by jointly exploiting both edge‐ and community‐level information, MSNR has the potential to yield novel insights into brain‐behavior relationships.

## 1 INTRODUCTION

Studying brain‐phenotype relationships in high‐dimensional connectomics is an active area of research in neuroscience (Bassett & Sporns, 2017; Bzdok et al., 2016). The advent of large neuroimaging datasets that provide measures of brain connectivity for unprecedented numbers of subjects has yielded novel insights into development and aging, cognition, and neuropsychiatric illnesses (Biswal et al., 2010; Bzdok & Yeo, 2017; Jernigan et al., 2016; Schumann et al., 2010; Van Essen et al., 2012). As the availability of datasets with rich neural, genetic, and behavioral measurements from large numbers of subjects continues to increase, there is a growing need for analytical tools that are tailored for the discovery of complex relationships between brain networks and phenotypes (Craddock, Tungaraza, & Milham, 2015; Varoquaux & Craddock, 2013; L. Wang, Durante, Jung, & Dunson, 2017; Zhang, Sun, & Li, 2018).

A typical brain network consists of hundreds of nodes that denote brain regions, and tens of thousands of edges that indicate connections between pairs of nodes (Rubinov & Sporns, 2009).The network can be viewed on the *micro‐scale*, *meso‐scale*, or *macro‐scale*. The *micro*‐scale of the network can be characterized by features of its edges. The *macro*‐scale of the network can be characterized by summary statistics such as characteristic path length and global efficiency (Rubinov & Sporns, 2009). The *meso‐scale* falls in between the micro‐scale and macro‐scale, and includes the communities that make up the network (Sporns & Betzel, 2016). A community refers to a collection of nodes that are highly connected to each other and have little connection to nodes outside of the community. Prior work has demonstrated that brain network architecture present on these different scales is associated with development and aging (Betzel et al., 2014; Gu et al., 2015; Power, Fair, Schlaggar, & Petersen, 2010), cognition (Bressler & Menon, 2010; Crossley et al., 2013; Park & Friston, 2013), and neuropsychiatric diseases (Bassett, Xia, & Satterthwaite, 2018; Braun et al., 2016; Fornito, Zalesky, & Breakspear, 2015; Grillon et al., 2013; Kernbach et al., 2018; Xia et al., 2018; Yu et al., 2019).

Despite increased appreciation that multi‐scale organization of the brain may be responsible for some of its major functions (Bassett & Siebenhühner, 2013; Betzel & Bassett, 2017), thus far, common strategies for studying the relationship between brain connectivity and phenotypes consider network features at a single scale (Craddock et al., 2015; Varoquaux & Craddock, 2013). For example, a popular single‐scale strategy focuses on group‐level comparisons of individual connections in brain networks (Bressler & Menon, 2010; Fornito et al., 2015; Grillon et al., 2013). This approach typically involves performing a statistical test on each network edge. While this procedure is easy to implement, several drawbacks limit its effectiveness (Bzdok & Ioannidis, 2019). Two main limitations are the need to account for multiple comparisons, and a lack of interpretability (Craddock et al., 2015; Varoquaux & Craddock, 2013). To achieve high power while minimizing the risk of false discovery, alternative edge‐based methods have been developed, such as the network‐based statistic (Zalesky, Fornito, & Bullmore, 2010) and multivariate distance matrix regression (Zapala & Schork, 2012). While these strategies have yielded important insights, they nonetheless focus exclusively on the micro‐scale without exploiting the multi‐scale information present in brain networks, often producing results that are difficult to interpret.

Given the importance of community structure in brain networks and its interpretability in the context of neural and cognitive computations (Betzel, Medaglia, & Bassett, 2018; Sporns & Betzel, 2016), it might be tempting to conduct a mass‐univariate analysis at the meso‐scale, considering *within*‐ and *between*‐community connectivity as the input features (Betzel et al., 2014; Braun et al., 2016; Gu et al., 2015; Yu et al., 2019). Such an approach dramatically reduces the dimensionality of the data, which in turn decreases the burden of multiple comparisons correction. A community‐based approach also has the added benefit of not having to deconstruct the connectivity matrix into vectors, as in an edge‐based approach, which inevitably disrupts the innate structure in the data. However, summarizing hundreds or thousands of edges as one single number to represent the connection within or between communities can be problematic. This is especially true for large communities such as the default mode network, whose edges are spatially distributed across the anterior and posterior portions of the brain (Raichle, 2015). Stated another way, extracting the mean connectivity at the community level risks mixing disparate signals.

In this paper, we introduce *Multi‐Scale Network Regression (MSNR)*, which simultaneously incorporates information across multiple scales in order to reveal associations between high‐dimensional connectomic data and phenotypes of interest. We first describe the MSNR model and introduce an algorithm to estimate the parameters. Next, we capitalize on one of the largest neurodevelopmental imaging cohorts, the Philadelphia Neurodevelopmental Cohort (PNC), to empirically assess the ability of MSNR in delineating brain connectivity patterns that are associated with a wide variety of phenotypes. Importantly, we conduct head‐to‐head comparisons between MSNR and common edge‐ and community‐based analyses that are based on single‐scale strategies, and show that MSNR achieves a balance between prediction performance and interpretability by considering information at multiple network scales.

## 2 METHODS

### 2.1 A statistical model for multi‐scale network regression

Given *n* subjects, let *A*^1^, …, *A*^*n*^ ∈ *R*^*p* × *p*^ denote the adjacency matrices corresponding to their brain connectivity networks, where *p* is the number of nodes. For instance, $A_{jj^{\prime }}^{i}$ could represent the Pearson correlation of the mean activation timeseries of two brain regions, a common measure of functional connectivity, between the *j*‐th and *j*^′^‐th nodes for the *i*‐th subject. Furthermore, we assume that the *p* nodes can be partitioned into *K* distinct communities *C*_1_, …, *C*_*K*_ that are known *a priori*: $\cup_{k=1}^{K}C_{k}=\left\{1,\ldots ,p\right\},C_{k}\cap C_{k^{\prime }}\neq \emptyset$ if *k* ≠ *k*^′^. The notation *j* ∈ *C*_*k*_ indicates that the *j*‐th node is in the *k*‐th community. Moreover, for each subject, *q* covariates have been measured, so that $X_{i}={\left(X_{i}^{1}\;X_{i}^{2}\ldots X_{i}^{q}\right)}^{T}\in {\mathbb{R}}^{q}$ is a covariate vector for the *i*‐th subject, *i* = 1, …, *n*.

In what follows, we consider the model

(1) $$A_{jj^{'}}^{i}=\Theta_{jj^{'}}+\sum_{f=1}^{q}\;X_{i}^{f}∙\left(\sum_{k=1}^{K}\;\sum_{k^{'}=1}^{K}\;\Gamma_{kk^{'}}^{f}1_{\left(j\in C_{k},j^{'}\in C_{k^{'}}\right)}\right)+{ɛ}_{jj^{'}}^{i},i=1,\ldots ,n,j,j^{'}=1,\ldots ,p,$$

Where ${ɛ}_{jj^{\prime }}^{i}$ is a mean‐zero noise term, and ${ɛ}_{jj^{\prime }}^{i}={ɛ}_{j^{\prime }j}^{i}$. Θ is a symmetric *p* × *p* matrix that summarizes the mean connectivity, across all of the subjects, of each pair of nodes, in the absence of covariates. Finally, for *f* = 1, …, *q*, Γ^*f*^ is a symmetric *K* × *K* matrix that quantifies the association between the *f*‐th feature and the functional connectivity between each pair of communities. For instance, a one‐unit increase in $X_{}^{f}$ is associated with a $\Gamma_{kk^{\prime }}^{f}$ increase in the mean functional connectivity between nodes in the *k*‐th and *k*^′^‐th communities.

We now define a *p* × *K* matrix *W* for which $W_{\mathrm{jk}}=1_{\left(j\in C_{k}\right)}$, where 1_(∙)_ denotes an indicator variable. As such, Equation (1) can be re‐written as

(2) $$\begin{array}{c} A^{i}=\Theta +\sum_{f=1}^{q}\;X_{i}^{f}∙\left(W\Gamma^{f}W^{T}\right)+{ɛ}^{i},\;i=1,\ldots ,n \end{array}.$$

In order to fit the Model (2), we make two assumptions about the structures of the unknown parameter matrices Θ and Γ^1^, …, Γ^*q*^.

Assumption 1: Θ *has low rank* (Leonardi et al., 2013; K. Li, Guo, Nie, Li, & Liu, 2009; Smith et al., 2015). That is, Θ = *VV*^*T*^ where *V* is a *p* × *d* matrix, for a small positive constant *d*. This means that the *p* nodes effectively reside in a reduced subspace of *d *dimensions. The mean connectivity between any pair of nodes is simply given by their inner product in this low‐dimensional subspace. Furthermore, to ensure identifiability, we assume that *Tr*(*W*^*T*^Θ*W*) = 0.

Assumption 2: Γ^1^, …, Γ^*q*^ *are sparse* (Eavani et al., 2015; Ng, Varoquaux, Poline, & Thirion, 2012; Xia et al., 2018). That is, most of their elements are *exactly* equal to zero. If $\Gamma_{kk^{\prime }}^{f}=0$, then the value of the *f*‐th feature is unassociated with the mean connectivity between nodes in the *k‐*th and *k*^′^‐th communities.

**Figure 1:**
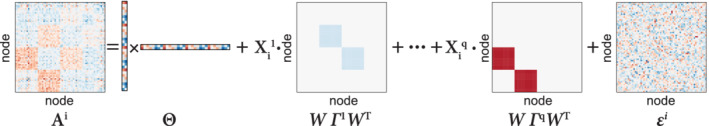
A schematic for Multi‐Scale Network Regression (MSNR). We developed a penalized multivariate approach for modeling brain networks that explicitly respects both edge‐ and community‐level information. We specified the MSNR model in Equation (2), which is visually represented here. Under the model, *A*^*i*^ is the connectivity matrix for the *i*‐th subject, Θ is a low‐rank matrix representing the mean connectivity across all subjects, Γ^1^, …, Γ^*q*^ are sparse matrices representing the community‐level connectivity associated with the covariates $\left(X_{i}^{1},\ldots ,X_{i}^{q}\right)$, and *ɛ*^*i*^ is the noise

We note that Assumption 1: is closely related to the random dot product graph model and similar models (Durante & Dunson, 2018; Durante, Dunson, & Vogelstein, 2017; Fosdick & Hoff, 2015; Tang, Athreya, Sussman, Lyzinski, & Priebe, 2017; Young & Scheinerman, 2007), whereas Assumption 2: is a standard sparsity assumption for high‐dimensional regression (Hastie, Tibshirani, & Friedman, 2008; Hastie, Tibshirani, Wainwright, Tibshirani, & Wainwright, 2015; Tibshirani, 1996). Under these two assumptions, a schematic of the Model (2) can be seen in Figure 1.

Model (2) is closely related to both the stochastic block model (Choi, Wolfe, & Airoldi, 2012) and the random dot product graph model (Young & Scheinerman, 2007). In particular, if Θ = 0, *q* = 1, and $X_{i}^{f}=1$ for *i* = 1, …, *n*, then Equation (2) reduces to a stochastic block model with known communities *C*_1_, …, *C*_*K*_. And if Γ^1^ = … = Γ^*q*^ = 0 and Assumption 1: holds, then Equation (2) reduces to a random dot product graph model. However, unlike those two models Equation (2) explicitly allows for the mean of the adjacency matrix to be a function of covariates, and effectively incorporates both edge‐ and community‐level network information.

### 2.2 Optimization problem

We now consider the task of fitting the Model (2), under Assumptions 1: and 2:. It is natural to consider the optimization problem

(3) $$\begin{array}{c} \underset{\Theta ,\Gamma^{1},...,\Gamma^{q}}{minimize}\left\{{\sum_{i=1}^{n}\;\left\|A^{i}-\left(\Theta +\sum_{f=1}^{q}\;X_{i}^{f}∙\left(W\Gamma^{f}W^{T}\right)\right)\right\|}_{F}^{2}+\lambda_{1}\mathrm{rank}\left(\Theta \right)+\lambda_{2}\sum_{f=1}^{q}\;{\left\|\Gamma^{f}\right\|}_{0}\right\}\; \end{array}$$

where the notation ${||\cdot ||}_{F}^{2}$ indicates the squared *Frobenius* norm of a matrix, that is, ${\left\|D\right\|}_{F}^{2}=\sum_{j=1}^{p}\sum_{j^{\prime }}^{p}D_{jj^{\prime }}^{2}$, and the notatiton ‖∙‖_0_ indicates the element‐wise cardinality (or *l*_0_ norm) of a matrix, that is, ${\left\|D\right\|}_{0}=\sum_{j=1}^{p}\sum_{j^{\prime }=1}^{p}1_{\left(D_{jj^{\prime }}\neq 0\right)}.$ In Equation (3), *λ*_1_ and *λ*_2_ are non‐negative tuning parameter values that control the rank of *Θ* and the sparsity of Γ^1^ = … = Γ^*q*^, respectively.

Unfortunately, due to the presence of the rank and *l*_0_ penalties, the optimization problem (3) is highly nonconvex, and no efficient algorithms are available to solve it. Therefore, in what follows, we will consider an alternative to Equation (3), which results from replacing the nonconvex rank and *l*_0_ penalties in Equation (3) with their convex relaxations. This leads to the optimization problem

(4) $$\begin{array}{c} \underset{\Theta ,\Gamma^{1},\ldots ,\Gamma^{q}}{\mathrm{minimize}}\left\{{\sum_{i=1}^{n}\left\|A^{i}-\left(\Theta +\sum_{f=1}^{q}X_{i}^{f}∙\left(W\Gamma^{f}W^{T}\right)\right)\right\|}_{F}^{2}+\lambda_{1}{\left\|\Theta \right\|}_{*}+\lambda_{2}\sum_{f=1}^{q}{\left\|\Gamma^{f}\right\|}_{1}\right\} \end{array}$$

In Equation (4), the notation ‖∙‖_*_ indicates the *nuclear norm* of a matrix, that is, the sum of its singular values (Bien & Witten, 2016; Fazel, 2002; Recht, Fazel, & Parrilo, 2010). The nuclear norm is a convex surrogate for the rank of a matrix. The notation ‖∙‖_1_ indicates the element‐wise *l*_1_ (or *lasso)* norm of a matrix, that is, ${\left\|D\right\|}_{1}=\sum_{j=1}^{p}\sum_{j^{\prime }=1}^{p}\left|D_{jj^{\prime }}\right|;$ this is a convex relaxation of the *l*_0_ norm (Hastie et al., 2015; Hastie et al., 2008; Tibshirani, 1996). In Equation (4), the non‐negative tuning parameters λ_1_ and λ_2_ encourage Θ and Γ^1^, …, Γ^*q*^ to be low‐rank and sparse, respectively.

Importantly, the optimization problem (4) is convex, and so fast algorithms are available to solve it for the global optimum. In Supporting Information, we have derived a block coordinate descent algorithm for solving Equation (4). Simulation studies indicated that MSNR behaved in the manner that was dependent on the signal‐to‐noise ratio and the observation‐to‐feature ratio, particularly in its ability to model underlying connectivity patterns (see Figure S1).

### 2.3 Philadelphia neurodevelopmental cohort

Resting‐state functional magnetic resonance imaging (rs‐fMRI) datasets were acquired as part of the Philadelphia Neurodevelopmental Cohort (PNC), a large community‐based study of brain development (Satterthwaite et al., 2014). In total, 1,601 participants completed the cross‐sectional neuroimaging protocol. Of these participants, 154 were excluded for meeting any of the following criteria: gross radiological abnormalities, history of medical problems that might affect brain function, history of inpatient psychiatric hospitalization, use of psychoactive medications at the time of data acquisition. Of the remaining 1,447 participants, 51 were excluded for low quality or incomplete FreeSurfer reconstruction of T1‐weighted images. Of the remaining 1,396 participants, 381 were excluded for missing rs‐fMRI, voxelwise coverage or excessive motion, which is defined as having an average framewise motion more than 0.20 mm and more than 20 frames exhibiting over 0.25 mm movement (using calculation from Jenkinson, Bannister, Brady, & Smith, 2002). These exclusions produced a final sample consisting of 1,015 youths (mean age 15.78, *SD* = 3.34; 461 males, and 554 females).

### 2.4 Imaging acquisition

Structural and functional imaging data were acquired on a 3T Siemens Tim Trio scanner with a 32‐channel head coil (Erlangen, Germany), as previously described (Satterthwaite et al., 2014, 2016). High‐resolution structural images were acquired in order to facilitate alignment of individual subject images into a common space. Structural images were acquired using a magnetization‐prepared, rapid‐acquisition gradient‐echo (MPRAGE) T1‐weighted sequence (*T*_*R*_ = 1, 810 ms; *T*_*E*_ = 3.51 ms; *FoV* = 180 × 240 mm; resolution 0.9375 × 0.9375 × 1 mm). Approximately 6 min of task‐free functional data were acquired for each subject using a blood oxygen level‐dependent (BOLD‐weighted) sequence (*T*_*R*_ = 3, 000 ms, *T*_*E*_ = 32 ms; *FoV* = 192 × 192 mm; resolution 3 mm isotropic; 124 volumes). Prior to scanning, in order to acclimatize subjects to the MRI environment and to help subjects learn to remain still during the actual scanning session, a mock scanning session was conducted using a decommissioned MRI scanner and head coil. Mock scanning was accompanied by acoustic recordings of the noise produced by gradient coils for each scanning pulse sequence. During these sessions, feedback regarding head movement was provided using the MoTrack motion tracking system (Psychology Software Tools, Inc., Sharpsburg, PA). Motion feedback was only given during the mock scanning session. In order to further minimize motion, prior to data acquisition subjects' heads were stabilized in the head coil using one foam pad over each ear and a third over the top of the head. During the resting‐state scan, a fixation cross was displayed as images were acquired. Subjects were instructed to stay awake, keep their eyes open, fixate on the displayed crosshair, and remain still.

### 2.5 Structural preprocessing

A study‐specific template was generated from a sample of 120 PNC subjects balanced across sex, race, and age using the buildTemplateParallel procedure in ANTs (Avants, Tustison, Song, et al., 2011). Study‐specific tissue priors were created using a multi‐atlas segmentation procedure (H. Wang et al., 2013). Next, each subject's high‐resolution structural image was processed using the ANTs Cortical Thickness Pipeline (Tustison et al., 2014). Following bias field correction (Tustison et al., 2010), each structural image was diffeomorphically registered to the study‐specific PNC template using the top‐performing SyN deformation (Klein et al., 2009). Study‐specific tissue priors were used to guide brain extraction and segmentation of the subject's structural image (Avants, Tustison, Wu, et al., 2011).

### 2.6 Functional preprocessing

Task‐free functional images were processed using the XCP Engine (Ciric et al., 2017), which was configured to execute a top‐performing pipeline for removal of motion‐related variance (Ciric et al., 2018). Preprocessing steps included (a) correction for distortions induced by magnetic field inhomogeneities using FSL's FUGUE utility, (b) removal of the four initial volumes of each acquisition, (c) realignment of all volumes to a selected reference volume using mcflirt (Jenkinson et al., 2002), (d) removal of and interpolation over intensity outliers in each voxel's time series using AFNI's 3Ddespike utility, (e) demeaning and removal of any linear or quadratic trends, and (f) co‐registration of functional data to the high‐resolution structural image using boundary‐based registration (Greve & Fischl, 2009). Confounding signals in the data were modeled using a total of 36 parameters, including the six framewise estimates of motion, the mean signal extracted from eroded white matter and cerebrospinal fluid compartments, the mean extracted from the entire brain, the derivatives of each of these nine parameters, and quadratic terms of each of the nine parameters and their derivatives. Both the BOLD‐weighted time series and the artefactual model time series were temporally filtered using a first‐order Butterworth filter with a passband between 0.01 and 0.08 Hz (Hallquist, Hwang, & Luna, 2013).

### 2.7 Network construction

**Figure 2:**
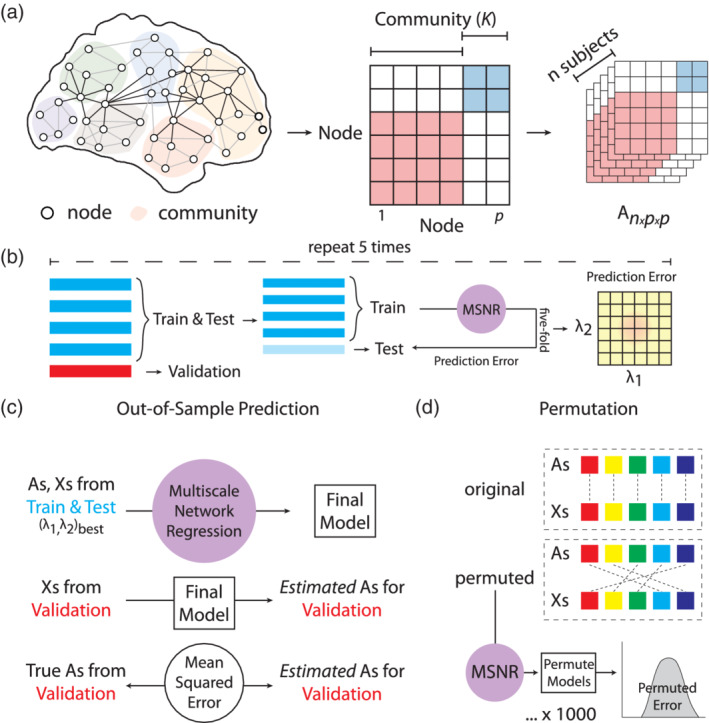
A schematic for MSNR model training and evaluation. (a) MSNR is designed to study the brain connectivity‐phenotype relationship by taking into account both edge‐ and community‐level information. The model takes in an *n* × *p* × *p* matrix, where *n* is the number of subjects and *p* is the number of nodes in each symmetric adjacency matrix. The nodes belong to *K* communities, determined *a priori*. (b) 20% (*n* = 202) of the total sample (*n* = 1, 015) were randomly selected as the left‐out validation data. We conducted five‐fold cross‐validation to select the values of the tuning parameters *λ*_1_ and *λ*_2_. These two parameters represent the nuclear norm penalty on the mean connectivity matrix (Θ) and the *l*_1_ norm of the community‐level connectivity‐covariate relationship matrices (Γ^1^, …, Γ^*q*^), respectively. This entire procedure was repeated five times. (c) The model was then trained using the tuning parameters determined in (b) on the rest 80% of the total data set (*n* = 813). Out‐of‐sample prediction error was then calculated as the *Frobenius* norm of the difference between the known and estimated connectivity matrices on the validation set. (d) We also evaluated the final model through a permutation procedure, where we disrupted the linkage between brain connectivity and covariate data to generate a null distribution of out‐of‐sample prediction error

We used a common parcellation of cortial and subcortical tissue into 264 regions (Power et al., 2011). The functional connectivity between any pair of brain regions was operationalized as the Fisher‐transformed Pearson correlation coefficient between the mean activation timeseries extracted from those regions. Connectomes were computed across all regions within a common parcellation with 264 nodes and 13 communities (Power et al., 2011). We encoded the pattern of functional connectivity in a formal network model in which nodes represent regions and edges represent functional connections. We assigned each region to one of 13 *a priori* communities (Power et al., 2011) that were delineated using the Infomap algorithm (Rosvall & Bergstrom, 2008) and replicated in an independent sample. We excluded 28 nodes that were not sorted into any community, therefore resulting in the final *p* = 236 and *K* = 13 (Figure 2a). This parcellation was selected for our analysis as it has been previously used for studying individual differences in brain connectivity, including those related to brain development (Gu et al., 2015; Satterthwaite et al., 2012), sex differences (Satterthwaite et al., 2015), and in‐scanner motion (Ciric et al., 2018).

### 2.8 Cross‐validation

We first randomly selected 20% of the total sample (*n* = 1, 015) to serve as the left‐out validation set (*n* = 202). We then performed five‐fold cross validation on the remaining 80% of the sample (*n* = 813) to select the values of the tuning parameters *λ*_1_ and *λ*_2_ for MSNR (James, Witten, Hastie, & Tibshirani, 2013, Figure 2b). To ensure the results were not due to any single random data split, we repeated the entire procedure above five times. The age distributions of subjects between training and validation sets across these five data partitions were similar to each other (Figure S2). In each fold, the independent variables (*X*_*n* × *q*_) were centered to a mean of zero and scaled by each column's standard deviation. The prediction error used in cross‐validation was the *Frobenius* norm of the difference between estimated and true connectivity matrices in the test set, ${\left\|A^{i}-{\hat{A}}^{i}\right\|}_{F}^{2}$ (Figure 2c). We ensured the prediction error was independent of the sample size by using the average prediction error over all subjects in the test set.

### 2.9 Permutation procedure

To estimate the distribution of prediction error under the null hypothesis of no association between functional connectivity and phenotype, we permuted the rows of the covariate matrix *X*_*n* × *q*_. For each permutation, we tuned *λ*_1_ and *λ*_2_ using cross‐validation, and calculated the prediction error in the left‐out validation set. The *p*‐value was defined to be the proportion of prediction errors among the 1,000 permuted datasets that are smaller than the prediction error on the observed data,

(5) $$\begin{array}{c} p_{\text{permutation}}=\frac{\sum_{1}^{1,000}1_{e_{i}\leq \bar{e}}}{1,000} \end{array}$$

where *e*_1_, …, *e*_1, 000_ denote the prediction errors on the 1,000 permuted data sets, and $\bar{e}$ denotes the prediction error on the original data. Here, 1_(*A*)_ is an indicator variable that equals 1 if the event *A*, and 0 otherwise.

### 2.10 Comparison to single‐scale approaches

We compared the performance of MSNR to two of the most commonly used single‐scale network regression strategies, namely the individual edge model (Grillon et al., 2013; Lewis, Baldassarre, Committeri, Romani, & Corbetta, 2009) and community mean model (Betzel et al., 2014; King et al., 2018; Yan et al., 2019; Yu et al., 2019). These two approaches have been commonly used to study connectivity‐phenotype relationships (Craddock et al., 2015; Varoquaux & Craddock, 2013) and differ primarily in terms of the scale of brain network examined (Figure S3). Details are as follows:

#### 2.10.1 Individual edge model

We vectorized the upper triangle of the adjacency matrix *A*^*i*^ for the *i*‐th subject, *i* = 1, …, *n*, in order to create a *n* × *p*(*p* − 1)/2 matrix. For each of the *p*(*p* − 1)/2 columns of this matrix, we fit a linear regression to model that column using three covariates: age, sex, and in‐scanner motion (Figure S3a). Specifically, we built a linear model for each edge in *R*, with the formula edge ~ age + sex + motion (Chen et al., 2016; Fjell et al., 2017; Wood, 2017; Xia et al., 2018, Figure S3b). We corrected the results for multiple comparisons using the false discovery rate (FDR, *q* < 0.05, Storey, 2002) and reshaped the *p*(*p* − 1)/2 columns to a *p* × *p* matrix for visualizing significant coefficients. To calculate out‐of‐sample prediction error, we used linear models fit for all edges. The prediction error was calculated in the same way as in MSNR.

#### 2.10.2 Community mean model

Community‐based linear models were built with mean *within‐* and *between‐*community connectivity as the dependent variables. The within‐community connectivity is defined as

(6) $$\begin{array}{c} \frac{\sum_{j,j^{\prime }\in C_{k}}A_{jj^{\prime }}^{i}}{\left|C_{k}\right|\times \left(\left|C_{k}\right|-1\right)} \end{array}$$

where $A_{jj^{\prime }}^{i}$ is the weighted edge strength between the node *j* and node *j*^′^, both of which belong to the same community *C*_*k*_, for the *i*‐th subject. The cardinality of the community assignment vector, |*C*_*k*_|, represents the number of nodes in the *k*‐th community. The between‐community connectivity is defined as

(7) $$\begin{array}{c} \frac{\sum_{j\in C_{k},j^{\prime }\in C_{k^{\prime }}}A_{jj^{\prime }}^{i}}{\left|C_{k}\right|\times \left|C_{k^{\prime }}\right|} \end{array}$$

where *C*_*k*_ and $C_{k^{\prime }}$ represent two different communities, and |*C*_*k*_| and $\left|C_{k^{\prime }}\right|$ are the number of nodes in each community, respectively.

By applying Equations 6, 7 to each subject, we created a $n\times \left[\frac{K\left(K-1\right)}{2}+K\right]$ matrix. For each of the $\frac{K\left(K-1\right)}{2}+K$ columns of this matric, we fit a linear model to predict that column using three covariates: age, sex, and in‐scanner motion. Similar to the edge‐based model, we built a linear model for each edge in R, with the formula community ~ age + sex + motion (Chen et al., 2016; Fjell et al., 2017; Wood, 2017; Xia et al., 2018 Figure S3b). We corrected the results for multiple comparisons using the false discovery rate (FDR, *q* < 0.05, Storey, 2002) and reshaped the $\frac{K\left(K-1\right)}{2}+K$ columns to a *K* × *K* matrix to visualize significant coefficients. To calculate out‐of‐sample prediction error, we used linear models fit for all communities. The prediction error was calculated in the same way as in MSNR.

### 2.11 Data and code availability

The data reported in this article have been deposited in database of Genotypes and Phenotypes (dbGaP): accession no. phs000607.v3.p2 [https://www.ncbi.nlm.nih.gov/projects/gap/cgi‐bin/study.cgi?study_id=phs000607.v3.p2].

An implementation of the algorithm is available at bitbucket.org/rshinohara/networkregression.

## 3 RESULTS

### 3.1 MSNR shows high accuracy in a large developmental sample

**Figure 3:**
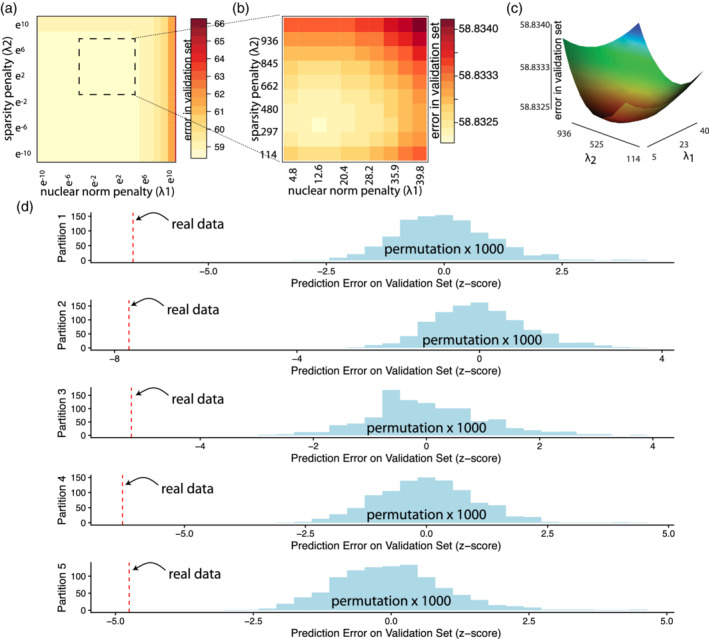
Tuning parameter selection and model evaluation of MSNR in a large neuroimaging dataset. (a) We used five‐fold cross‐validation in each data partition to estimate the test prediction error associated with various values of *λ*_1_ and *λ*_2_. The matrix here represents the average error across five different data partition. (b) After the initial search, we repeated the search on a finer scale, focusing on the range of *λ*_1_ *and* *λ*_2_ indicated by the dashed‐line box. (c) As visualized, no boundary effect was observed in the grid search, revealing a smooth convex landscape for the objective, with warmer color indicating lower prediction error. (d) In each data partition, a permutation procedure showed that the MSNR fit to the original data significantly outperformed that to the permutated data with regards to prediction error on the validation set (*p* < .001). Consistent across five data partitions, the prediction error was consistently multiple standard deviations (*z*‐score) below the mean of the null distributions

We applied MSNR to data from the Philadelphia Neurodevelopmental Cohort (PNC) (Satterthwaite et al., 2014) in order to delineate meaningful brain‐phenotype relationships. In total, we studied *n* = 1, 015 participants aged 8–22, who completed resting state functional neuroimaging as part of the PNC. We constructed functional connectivity matrices from a commonly‐used parcellation scheme (=236 nodes) and community membership assignment (*K* = 13 communities; Power et al., 2011, Figure 2a). We first randomly selected 20% of the total sample as the left‐out validation set (*n* = 202), with which we assessed the prediction performance of all subsequent models Figure 2b). The prediction performance was defined as the *Frobenius* norm of the difference between the observed and estimated adjacency matrices in the validation set (Figure 2c). For this proof‐of‐concept empirical study, we examined the association of functional connectivity with age, sex, and in‐scanner motion. On the remaining 80% of the observations, we selected tuning parameters, λ_1_ and λ_2_, through five‐fold cross‐validation (Figure 2b). We iteratively refined the cross‐validation grid (Figure 3a,b) in order to obtain the best possible tuning parameter values. Importantly, no boundary effect was observed in any of the iterations during successive grid searches, revealing a smooth convex landscape for the objective (Figure 3c).

We subsequently evaluated the model's out‐of‐sample prediction error on the validation set. The prediction error on the unseen data was comparable to the average error in the cross‐validation procedure, indicating MSNR did not overfit to the training data. In addition, to determine the statistical significance of the model, we performed a permutation test to compare the model's prediction error to the distribution of prediction error under the null hypothesis of no association between brain networks and the predictors (Figure 3d). Specifically, we permuted the rows of the covariate data matrix 1,000 times. In each permutation, we disrupted the linkage between functional connectivity and phenotypes, while preserving the covariance structure of the covariates. We repeated the process of selecting tuning parameter values by cross‐validation. Using these permuted data, we created a null distribution of prediction error. We then compared the true MSNR prediction error against this null distribution to estimate the *p*‐value. We found that MSNR fit to the originally data outperformed any of the 1,000 null model when there was no linkage between covariates and connectivity (*p* < .001).

### 3.2 MSNR recapitulates known individual differences in functional connectivity

**Figure 4:**
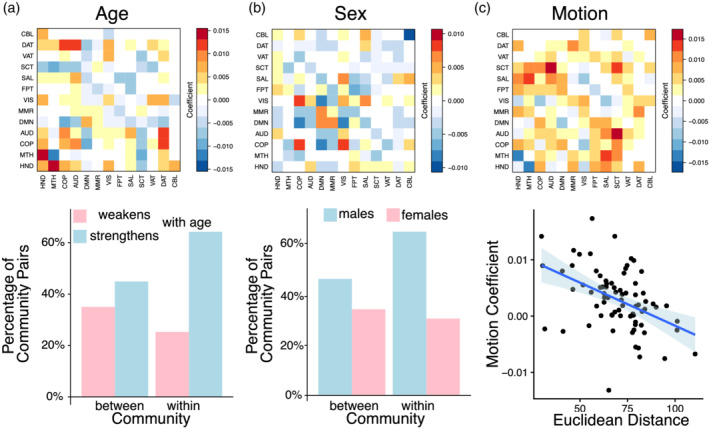
MSNR describes meaningful individual differences in brain connectivity. Top row represents the coefficient matrix Γ for each of the three phenotypes modeled in the MSNR. (a) We counted the number of positive and negative coefficients related to age. More within‐community, rather than between‐community, connectivity strengthened as the age increased. Conversely, more between‐community, rather than within‐community, connectivity weakened over age. (b) Stronger within‐community than between‐community connectivity was more representative of male functional brain networks, whereas stronger between‐community than within‐community connectivity was more representative of female functional brain networks. (c) Coefficient for in‐scanner motion was negatively correlated with the average Euclidean distance between communities (*p* < .001)

Next, we investigated connectivity‐phenotype relationships are summarized in the matrices Γ^1^, Γ^2^, and Γ^3^ in the MSNR model. These coefficient matrices corresponded to the multivariate connectivity associated with age, sex, and motion, respectively (Figure 4). Of note, these matrices were relatively sparse, with 20.1, 19.5, and 16.6% entries exactly equaling to zero. Among the nonzero entries, many were previously reported in the literature. For example, stronger DMN connectivity was associated with age (Bluhm et al., 2008; Staffaroni et al., 2018) and with female sex (Bluhm et al., 2008). To summarize the multivariate patterns extracted by MSNR, We counted the number of positive and negative coefficients within each estimated matrix. These represent, respectively, positive and negative associations between community membership and age, sex, and in‐scanner motion (Figure 4). Consistent with the previous literature (Gu et al., 2015; Satterthwaite et al., 2013), we found that as age increased, there were more within‐community, rather than between‐community connectivity, that strengthened with age (Figure 4a). Conversely, as age increased, there were more between‐community, rather than within‐community connectivity, that weakened with age. This pattern of results suggests that functional brain networks tend to segregate during normative brain development. Replicating findings from a previous report using mass‐univariate analyses (Satterthwaite et al., 2015), here we observed that stronger within‐community connectivity, rather than between‐community, was more representative of functional brain networks in males; whereas stronger between‐community connectivity, rather than within‐community, was more representative of functional brain networks in females (Figure 4b). Finally, following on prior studies, we evaluated the degree to which the association between in‐scanner motion and connectivity varies by inter‐node distance, defined as the Euclidean distance between two spherical brain parcellations in the MNI space (Brett, Johnsrude, & Owen, 2002, Figure 4c). As expected, the MSNR coefficients for in‐scanner motion in relation to functional connectivity were negatively correlated with the distances between pairs of communities. In other words, when two brain regions were close together, the presence of in‐scanner motion was typically associated with an increase in their connectivity. This finding is consistent with prior reports that in‐scanner motion induces a distance‐dependent bias in estimation of functional connectivity (Ciric et al., 2017; Satterthwaite et al., 2012). Of note, we verified that the Θ matrix was indeed low‐rank (Figure S4).

### 3.3 Comparison with typical mass‐univariate single‐scale strategies

**Figure 5:**
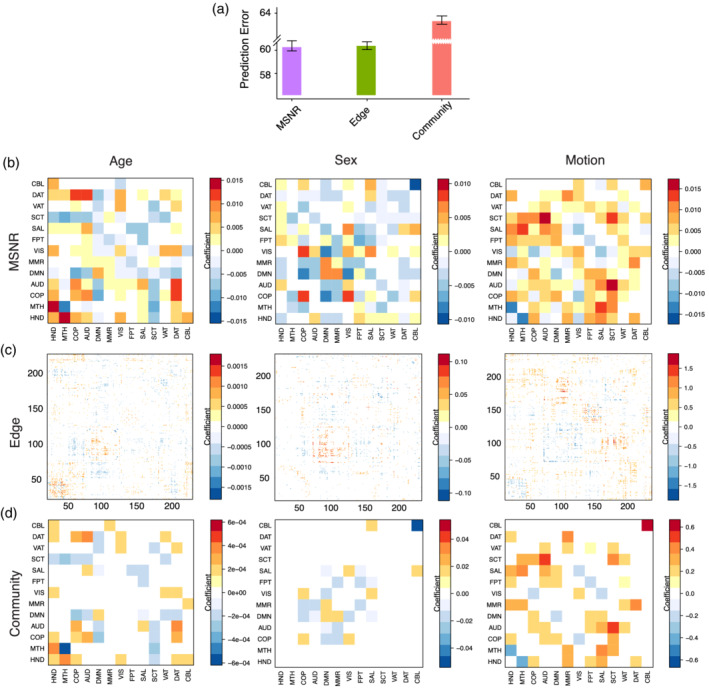
MSNR achieves a balance between out‐of‐sample prediction performance and model interpretability compared to common single‐scale mass‐univariate approaches. (a) We compared out‐of‐sample prediction performance of MSNR to common single‐scale mass univariate analysis such as edge‐ and community‐based methods. Among the three methods, the community‐based approach had the highest prediction error. In contrast, MSNR had similar prediction error as the edge‐based approach. Error bar represents the standard deviation across five different data partitions. (b) MSNR coefficients in Γ describe the multivariate connectivity‐phenotype relationships. These correspond to age, sex, and in‐scanner motion, respectively. Results from single‐scale models were visualized in (c) for edge‐based and in (d) for community‐based approaches. Multiple comparisons were corrected using FDR

Next, we compared MSNR to common single‐scale mass‐univariate approaches that make use of linear models at the edge‐level or the community‐level (Figure 5). We computed the out‐of‐sample performances of the two single‐scale approaches using the left‐out validation set. The prediction error of the community‐based model on the validation set was poor, whereas that of the edge‐based model was similar to MSNR (Figure 5a). Our estimation of prediction error of edge‐ and community‐based models were likely to be overly optimistic, since we used all fitted models for the purpose of out‐sample prediction.

Next, we examined the interpretability of coefficients obtained in each model after applying FDR correction to control for multiple comparisons in single‐scale approaches (Storey, 2002). We found that while the edge‐based model and MSNR achieved similar out‐of‐sample prediction, coefficients estimated in MSNR (Figure 5b) were more interpretable than the coefficients from edge‐based models (Figure 5c). The number of coefficients in edge‐based models for each covariate exceeded that of MSNR by three orders of magnitude. On the other hand, at the expense of higher prediction error, community‐based models exhibited a level of interpretability that was similar to that exhibited by MSNR (Figure 5d).

## 4 DISCUSSION

In the past decade, the neuroscience community has begun to complement the study of localized regions of the brain toward studying inter‐regional relationships, or connectivity (Bassett & Sporns, 2017; Bzdok et al., 2016). The association of network architecture with development and aging throughout the lifespan (Betzel et al., 2014; Gu et al., 2015; Power et al., 2010), cognition (Bressler & Menon, 2010; Crossley et al., 2013; Park & Friston, 2013), and neuropsychiatric disorders (Bassett et al., 2018; Xia et al., 2018; Yu et al., 2019) is of profound interest to the burgeoning network neuroscience literature. These brain‐phenotype associations can be studied on the scale of individual edges (*micro*‐scale), communities (*meso*‐scale), or the network as a whole (*macro*‐scale), with most existing approaches for analyzing networks, such as mass‐univariate analyses, operate on a single scale.

In recent years, interest has centered on multi‐scale modeling approaches (Jenatton et al., 2012; Y. Li et al., 2011, 2013), which aim to integrate information across homogeneous regions in the brain while still modeling data on finer scales. These methods have mainly focused on the problem of smoothing without prior knowledge of anatomical or functional parcellations of the brain, and have been adapted for both classification (Romberg, Choi, Baraniuk, & Kingbury, 2000) and regression (Y. Li et al., 2011) as well as in longitudinal settings (Y. Li et al., 2013).

Building upon this recent work, we developed MSNR to study relationships between high‐dimensional brain networks and variables of interest. Specifically, our proposal modeled the connectivity matrix for each subject by integrating both *micro*‐ and *meso*‐scale network information. By applying a low‐rank assumption to the mean connectivity network (Leonardi et al., 2013; K. Li et al., 2009; Smith et al., 2015) and a sparsity assumption to the community‐level network (Meunier, Lambiotte, & Bullmore, 2010; Newman, 2006; Xia et al., 2018), we substantially decreased the number of parameters and encouraged the detection of interpretable brain‐phenotype relationships.

Leveraging a large neuroimaging dataset of over 1000 youth, we demonstrated that MSNR recapitulated known individual differences in functional connectivity, including those related to development (Gu et al., 2015; Satterthwaite et al., 2013), sex differences (Satterthwaite et al., 2015), and in‐scanner motion (Ciric et al., 2017; Satterthwaite et al., 2012). Notably, compared to common single‐scale mass‐univariate regression methods, MSNR achieved a balance between prediction performance and model complexity, with improved interpretability. Together, MSNR represents a new method for identifying individual differences in high‐dimensional brain networks.

Several limitations of the MSNR approach should be noted. First, the term “scale” does not have a single definition. In fact, as pointed out by Betzel and Bassett (2017), scale can represent at least three different entities depending on the context: multi‐scale topological structure, multi‐scale temporal structure, and multi‐scale spatial structure. In MSNR, we only considered multi‐scale topological structure. Incorporating additional information from multiple scales beyond network topology will likely generate more nuanced and richer models for brain networks. Second, while we carefully conducted a permutation test to assess the statistical significance of the entire model, we did not provide an inferential procedure for determining the association between brain networks and each variable of interest. In particular, MSNR makes no claim of statistical significance for the coefficients in the matrices Γ^1^, …, Γ^q^, which describe the community‐level relationships with the covariates. Due to the inclusion of penalty terms in the MSNR framework, making such inferential statements is a challenging open problem.

In summary, by explicitly modeling variability at the edge and community levels, we developed a multi‐scale network regression approach that achieved a balance between the trade‐off of prediction and model complexity, potentially offering enhanced interpretability. Empirically, we demonstrated its advantages over alternative methods and illustrated its ability to uncover meaningful signals in a large neuroimaging dataset. Approaches such as MSNR have the potential to yield novel insights into brain‐behavior relationships that incorporate realistic multi‐scale network architecture.

## CONFLICT OF INTEREST

R.T.S. received consulting income from Genentech/Roche and income for editorial duties from the American Medical Association and Research Square. All other authors declare no conflict of interest.

## Supporting information


**Appendix** **S1**: Supporting Information

## Data Availability

The data reported in this paper have been deposited in database of Genotypes and Phenotypes (dbGaP): accession no. phs000607.v3.p2 [https://www.ncbi.nlm.nih.gov/projects/gap/cgi‐bin/study.cgi?study_id=phs000607.v3.p2]. An implementation of the algorithm is available at bitbucket.org/rshinohara/networkregression.
