# Supplementary material for: Multi‐scale network regression for brain‐phenotype associations
Source: Hum Brain Mapp. 2020 Mar 26;41(10):2553–66. doi: 10.1002/hbm.24982 (PMC7383128; doi:10.1002/hbm.24982)
Supplement: Supplementary file 1 — Appendix S1: Supporting Information [file HBM-41-2553-s001.docx]

**Supplementary Information**

1. Statistical Methodology of MSNR
   1. Block Coordinate Descent Algorithm

The optimization problem of Eq. 4 in the main manuscript is

$$\underset{\Theta, \Gamma^{1},\ldots,\Gamma^{q}}{\mathrm{minimize}} \left\{ {\sum_{i=1}^{n} \left\| A^{i}-\left( \Theta+\sum_{f=1}^{q} X_{i}^{f}\cdot\left( W\Gamma^{f}W^{T} \right) \right) \right\|}_{F}^{2}+\lambda_{1}\left\| \Theta\right\|_{*}+\lambda_{2}\sum_{f=1}^{q} \left\| \Gamma^{f} \right\|_{1} \right\} . \left( 1 \right)$$

We now derive a block coordinate descent algorithm for solving (4) (Bien & Witten, 2016; Friedman, Hastie, Höfling, & Tibshirani, 2007; Hastie, Tibshirani, & Friedman, 2008; Tseng, 2001). Roughly speaking, we will cycle through the parameters $\Theta,\Gamma^{1}=\ldots=\Gamma^{q}$, and minimize the objective (4) with respect to each one in turn, holding all others fixed. Because the loss function is differentiable and the penalties are separable with respect to each block of parameters, this approach is guaranteed to yield the global optimum. The algorithm is as follows:

1. Initialize a $p\times p$ matrix $\hat{\Theta}$, and $K\times K$ matrices $\hat{\Gamma}^{1},\ldots, \hat{\Gamma}^{q}.$
2. Iterate until convergence:
   1. Update $\Theta$ by minimizing (4) with respect to $\Theta$, holding $\hat{\Gamma}^{1},\ldots, \hat{\Gamma}^{q}$ fixed:

$$\begin{aligned} \hat{\Theta}\leftarrow\left\{ {\sum_{i=1}^{n} \left\| A^{i}-\left( \Theta+\sum_{f=1}^{q} X_{i}^{f}\cdot\left( W\hat{\Gamma}^{f}W^{T} \right) \right) \right\|}_{F}^{2}+\lambda_{1}\left\| \Theta\right\|_{*} \right\}. \#\left( 2 \right) \end{aligned}$$

- 1. For $f=1,\ldots,q$, update $\Gamma^{f}$ by minimizing (4) with respect to $\Gamma^{f}$, holding $\hat{\Theta}$ and $\hat{\Gamma}^{1},\ldots, {\hat{\Gamma}^{f-1},\hat{\Gamma}^{f+1},\ldots,\hat{\Gamma}}^{q}$ fixed:

$$\begin{aligned} \hat{\Gamma}^{f}\leftarrow\left\{ {\sum_{i=1}^{n} \left\| A^{i}-\left( \hat{\Theta}+\sum_{f^{'}\neq f} X_{i}^{f^{'}}\cdot\left( W{\hat{\Gamma}^{f^{'}}W}^{T} \right)+X_{i}^{f}\cdot\left( W\Gamma^{f}W^{T} \right) \right) \right\|}_{F}^{2}+\lambda_{2}\left\| \Gamma^{f} \right\|_{1} \right\}. \# \end{aligned}\left( 3 \right)$$

Both (5) and (6) are convex optimization problems, for which closed form solutions are available, as detailed in the following propositions. These propositions make use of the soft-thresholding operator, defined as

$$\begin{aligned} S\left( a,b \right)=\left( \left| a \right|-b,0 \right) sign\left( a \right), \#\left( 4 \right) \end{aligned}$$

and applied element-wise to the elements of a matrix.

***Proposition 1.*** Define

$$\tilde{A}^{i}\equiv A^{i}-\sum_{f=1}^{q} X_{i}^{f}\cdot\left( W\hat{\Gamma}^{f}W^{T} \right),$$

and let $\mathrm{UD}V^{T}$denote the singular value decomposition of $\frac{1}{n}\sum_{i=1}^{n} \tilde{A}^{i}$: that is, $\frac{1}{n}\sum_{i=1}^{n} \tilde{A}^{i}=UDV^{T}$, where $U$ and $V$ are $p\times p$ matrices, $U^{T}U=UU^{T}=V^{T}V=VV^{T}=I$, and $D$ is a diagonal matrix with non-negative elements on the diagonal elements on the diagonal. Then, the solution to the optimization problem (5) is

$$\hat{\Theta}=US\left( D,\frac{\lambda_{1}}{2n} \right)V^{T},$$

where the soft-thresholding operator defined in (7) is applied element-wise.

Let $p_{k}\equiv\left| C_{k} \right|$, the cardinality of the $k$th community; note that $\sum_{k=1}^{K} p_{k}=p$.

***Proposition 2.*** Let $W_{j}$ denote the $j$th row of the matrix $W$. For $f=1, \ldots, q,$ define

$\bar{A}_{jj^{'}}\equiv A_{jj^{'}}^{i}-\bar{\Theta}_{jj^{'}}-\sum_{f^{'}\neq f} X_{i}^{f^{'}}\cdot\left( W_{j}^{T}\hat{\Gamma}^{f^{'}}W_{j^{'}} \right)$,

$$\tilde{y}_{kk^{'}}^{f}\equiv\frac{\sum_{j\in C_{k}} \sum_{j^{'}\in C_{k}} \sum_{i=1}^{n} \bar{A}_{jj^{'}}\cdot X_{i}^{f}}{\sum_{i=1}^{n} \left( X_{i}^{f} \right)^{2}p_{k}p_{k^{'}}},$$

and

$$\tilde{\lambda}_{kk^{'}}^{f}\equiv\frac{\lambda_{2}}{\sum_{i=1}^{n} \left( X_{i}^{f} \right)^{2}p_{k}p_{k^{'}}} .$$

Then, the solution to the optimization problem (6) is of the form

$$\tilde{\Gamma}_{kk^{'}}^{f}\equiv S\left( \tilde{y}_{kk^{'}}^{f},\frac{\tilde{\lambda}_{kk^{'}}^{f}}{2} \right).$$

Proofs of Propositions 1 and 2 are provided below.

*Proof of Proposition 1.*

Given the definition of $\tilde{A}_{i}$, (5) reduces to the optimization problem

$$\begin{aligned} \underset{\Theta}{\mathrm{minimize}} \left\{ \sum_{i=1}^{n} \left\| \Theta-\tilde{A}_{i} \right\|_{F}^{2}+\lambda_{1}\left\| \Theta\right\|_{*} \right\}. \#\left( 11 \right) \end{aligned}$$

We notice that

$$\sum_{i=1}^{n} \begin{aligned} \left\| \Theta-\tilde{A}^{i} \right\|_{F}^{2}=n\left( \left\| \Theta\right\|_{F}^{2}-2trace\left[ \Theta\left( \sum_{i=1}^{n} \frac{\tilde{A}^{i}}{n} \right) \right] \right)+C \\ =n\left\| \Theta-\sum_{i=1}^{n} \frac{\tilde{A}^{i}}{n} \right\|_{F}^{2}+C^{'}, \end{aligned}$$

where $C$ and $C^{'}$ are not a function of $\Theta$. Therefore, (11) can be re-written as

$$\begin{aligned} \underset{\Theta}{\mathrm{minimize}} \left\{ \left\| \Theta-\sum_{i=1}^{n} \frac{\tilde{A}^{i}}{n} \right\|_{F}^{2}+\lambda_{1}\left\| \Theta\right\|_{*} \right\}. \#\left( 12 \right) \end{aligned}$$

The result follows directly from Lemma 1 of Mazumder et al. (2010).

*Proof of Proposition 2.*

We wish to solve the problem

$$\underset{\Gamma^{f}}{\mathrm{minimize}} \left\{ \sum_{i=1}^{n} \left\| A^{i}-\left( \hat{\Theta}+\sum_{f^{'}\neq f} X_{i}^{f^{'}}\cdot\left( W\hat{\Gamma}^{f^{'}}W^{T} \right)+X_{i}^{f}\cdot\left( W\Gamma^{f}W^{T} \right) \right) \right\|_{F}^{2}+\lambda_{2}\left\| \Gamma^{f} \right\|_{1} \right\}.$$

Given the definition of $\bar{A}^{i}$, this amounts to solving

$$\begin{aligned} \underset{\Gamma^{f}}{\mathrm{minimize}} \begin{aligned} \left\{ \sum_{i=1}^{n} \left\| \bar{A}^{i}-X_{i}^{f}\cdot\left( W\Gamma^{f}W^{T} \right) \right\|_{F}^{2}+\lambda_{2}\left\| \Gamma^{f} \right\|_{1} \right\}. \# \\ \end{aligned} \#\left( 13 \right) \end{aligned}$$

So, for $k=1,\ldots, K$ and $k^{'}=1,\ldots, K,$ we must solve the problem

$$\begin{aligned} \underset{\Gamma_{kk^{'}}^{f}}{\mathrm{minimize}} \left\{ \sum_{i=1}^{n} \sum_{j\in C_{k}} \sum_{j^{'}\in C_{k^{'}}} \left( \bar{A}_{jj^{'}}^{i}-X_{i}^{f}\Gamma_{kk^{'}}^{f} \right)^{2}+\lambda_{2}\left| \Gamma_{kk^{'}}^{f} \right| \right\}. \#\left( 14 \right) \end{aligned}$$

And note that

$$\sum_{i=1}^{n} \sum_{j\in C_{k}} \sum_{j^{'}\in C_{k^{'}}} \left( \bar{A}_{jj^{'}}^{i}-X_{i}^{f}\Gamma_{kk^{'}}^{f} \right)^{2}+\lambda_{2}\left| \Gamma_{kk^{'}}^{f} \right|=C-2\left( \sum_{i=1}^{n} \sum_{j\in C_{k}} \sum_{j^{'}\in C_{k^{'}}} {\bar{A}_{jj^{'}}^{i}X_{i}^{f}} \right)\Gamma_{kk^{'}}^{f}+p_{k}p_{k^{'}}\sum_{i=1}^{n} \left( X_{i}^{f} \right)^{2}\left( \Gamma_{kk^{'}}^{f} \right)^{2},$$

where $C$ is not a function of $\Gamma^{f}$. So the problem of interest amounts to minimizing

$$-2\frac{\left( \sum_{i=1}^{n} \sum_{j\in C_{k}} \sum_{j^{'}\in C_{k^{'}}} {\bar{A}_{jj^{'}}^{i}X_{i}^{f}} \right)}{p_{k}p_{k^{'}}\sum_{i=1}^{n} \left( X_{i}^{f} \right)^{2}}\Gamma_{kk^{'}}^{f}+\left( \Gamma_{kk^{'}}^{f} \right)^{2}+\frac{\lambda_{2}}{p_{k}p_{k^{'}}\sum_{i=1}^{n} \left( X_{i}^{f} \right)^{2}}\left| \Gamma_{kk^{'}}^{f} \right|$$

with respect to $\Gamma_{kk^{'}}^{f}$. Thus, the minimizer is

$$S\left( \frac{\sum_{i=1}^{n} X_{i}^{f}\sum_{j\in C_{k}} \sum_{j^{'}\in C_{k^{'}}} \bar{A}_{jj^{'}}^{i}}{p_{k}p_{k^{'}}\sum_{i=1}^{n} \left( X_{i}^{f} \right)^{2}},\frac{\lambda_{2}}{{2p}_{k}p_{k^{'}}\sum_{i=1}^{n} \left( X_{i}^{f} \right)^{2}} \right).$$

1. Simulation Studies

We used the Brain Connectivity Toolbox (Rubinov & Sporns, 2009) to create random modular small-world adjacency matrices of dimension $p\times p$ with specified community assignments ($K=4$) representing the edge-level information. These adjacency matrices were then used as the ground truth mean connectivity in stimulated data, $\Theta_{0}$. We also created sparse $K\times K$ matrices $\Gamma_{0}^{1},\ldots, \Gamma_{0}^{q}$, representing ground truth community-level brain-phenotype relationships. We constructed the ground truth adjacency matrix for the $i$-th observation as $A_{0}^{i}=\Theta_{0}+\gamma\sum_{f=1}^{q} X_{i}^{f}\cdot(W\Gamma_{0}^{f}W^{T})$, where the elements $X_{i}^{f}$were independently generated from a normal distribution, scaled by a factor of $\gamma$ to represent the effect size. Then, we generated the observed connectivity matrix $A^{i}=A_{0}^{i}+\epsilon_{i}$ for a noise matrix $\epsilon_{i}$.

We created synthetic network data with varying characteristics, such as different numbers of nodes ($p\in\left\{ 32, 64, 128 \right\}$), sample sizes ($n\in\left\{ 50, 100, 150 \right\}$), effect sizes ($\gamma\in\left\{ 0, 0.1, 0.5, 1 \right\}$), and noise levels ($\epsilon\in\left\{ 0, 0.1, 0.5, 1 \right\}$), for a total of 108 combinations of these parameters. For each combination, we generated three equally-size sets, for training, testing, and validation. Tuning parameters $\lambda_{1}$ and $\lambda_{2}$ were selected using the training and testing sets, and the out-of-sample prediction error was computed on the validation set.

We found that MSNR achieved the lowest out-of-sample prediction error when the ratio between the number of subjects and the number of nodes was the largest ($n=150$, $p=32$) (**Supplementary Figure 1**). In addition, the amount of noise impacted MSNR's prediction performance in a graded fashion, with a three-fold difference between the lowest noise level (0.1) and the highest noise level (1). In contrast, MSNR was less sensitive to the varying levels of $\gamma$, which represents the effect size of the community level relationship of the covariates. These results were to be expected, as when the model is well specified in the sense that the data is generated according to the model, the more observations available or the smaller the noise means one can estimate the model parameters more accurately.

**References**

Bien, J., & Witten, D. (2016). Penalized estimation in complex models. In *Handbook of Big Data* (pp. 285–299).

Friedman, J., Hastie, T., Höfling, H., & Tibshirani, R. (2007). Pathwise coordinate optimization. *The Annals of Applied Statistics*, *1*(2), 302–332. https://doi.org/10.1214/07-AOAS131

Hastie, T., Tibshirani, R., & Friedman, J. (2008). *The Elements of Statistical Learning* (2nd ed.). Stanford, CA: Springer.

Mazumder, R., Hastie, T., Edu, H., Tibshirani, R., Edu, T., & Jaakkola, T. (2010). *Spectral Regularization Algorithms for Learning Large Incomplete Matrices*. *Journal of Machine Learning Research* (Vol. 11).

Rubinov, M., & Sporns, O. (2009). Complex network measures of brain connectivity : Uses and interpretations. *NeuroImage*, *52*(3), 1059–1069. https://doi.org/10.1016/j.neuroimage.2009.10.003

Tseng, P. (2001). Convergence of a Block Coordinate Descent Method for Nondifferentiable Minimization. *Journal of Optimization Theory and Applications*, *109*(3), 475–494. https://doi.org/10.1023/A:1017501703105


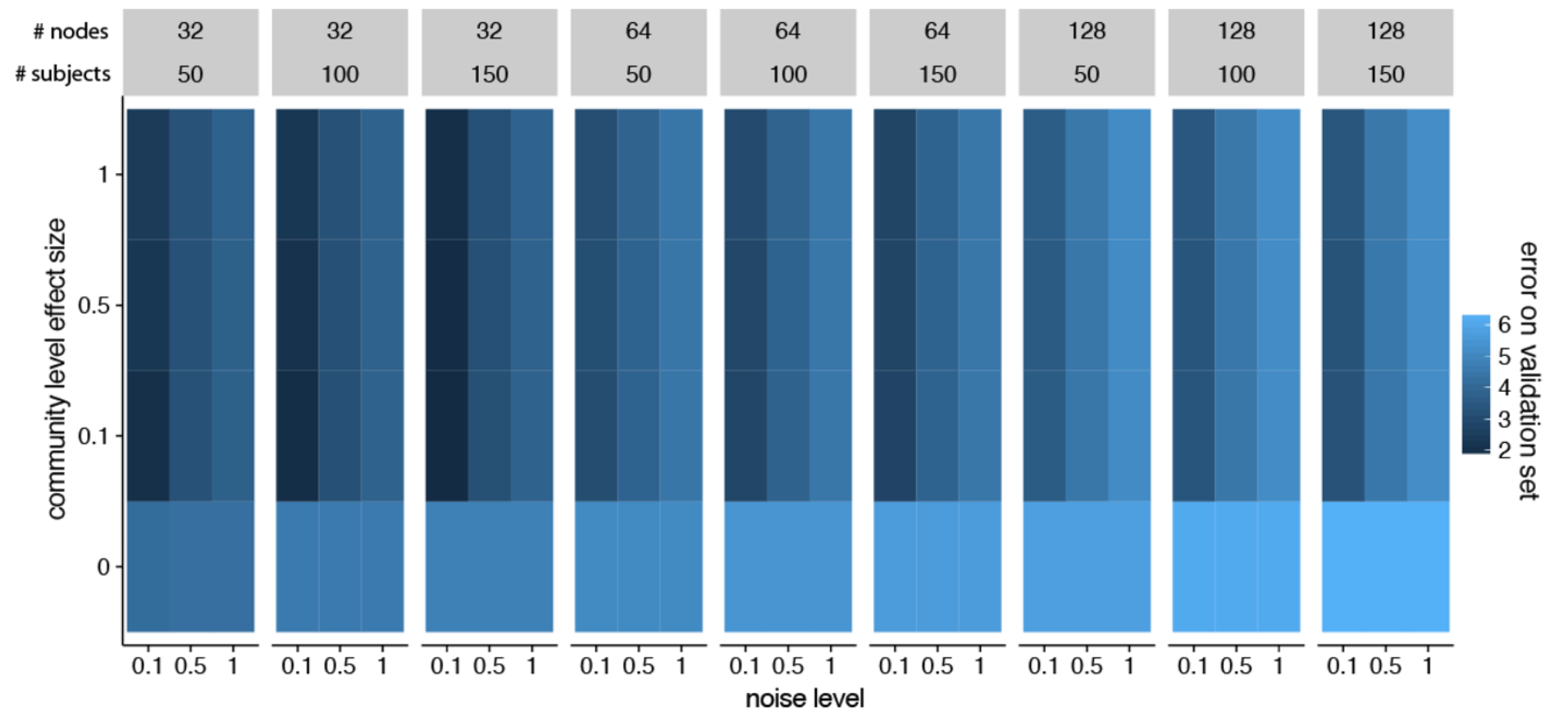


**Supplementary Figure 1 | Performance of MSNR in a simulation study.** We simulated data with varying numbers of observations ($n$) and nodes ($p$), effect size ($\gamma$) of $\Gamma^{1}, ...\Gamma^{q}$, and noise levels ($\varepsilon$). As expected, the performance of MSNR improved as the ratio of $n$ to $p$ increased, and as the signal-to-noise ratio increased. In contrast, MSNR was less sensitive to the varying levels of$\gamma$, which represents the effect size of the community level relationship of the covariates.


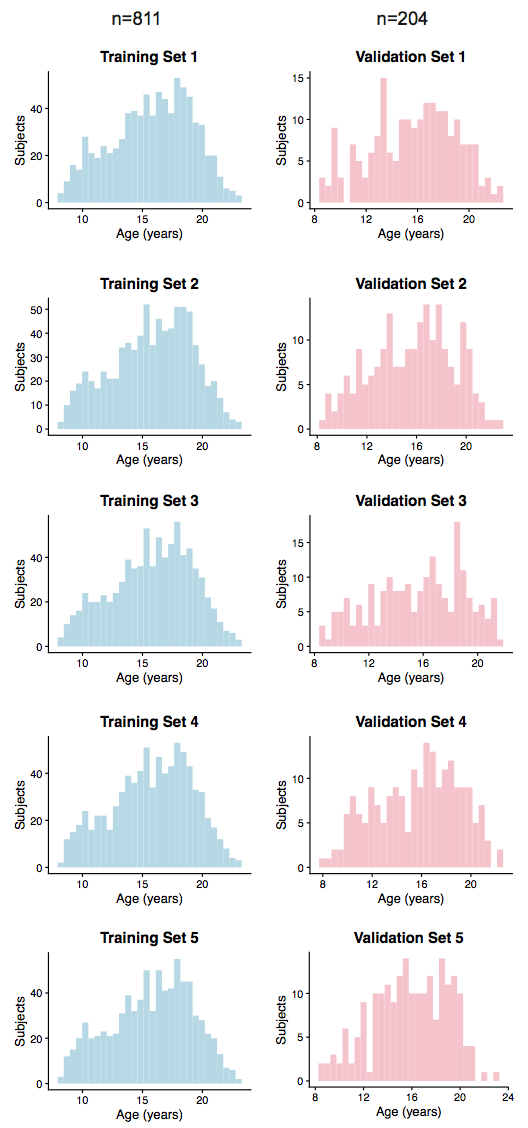


**Supplementary Figure 2 | Age distributions of the training and validation set from five data partitions.** In each experiment, we randomly selected 20% (n=204) from the total sample as the left-out validation set, and the remaining 80% (n=811) as the training set. The age distributions were similar between training and validation set in each data partition, as well as across different data partitions.


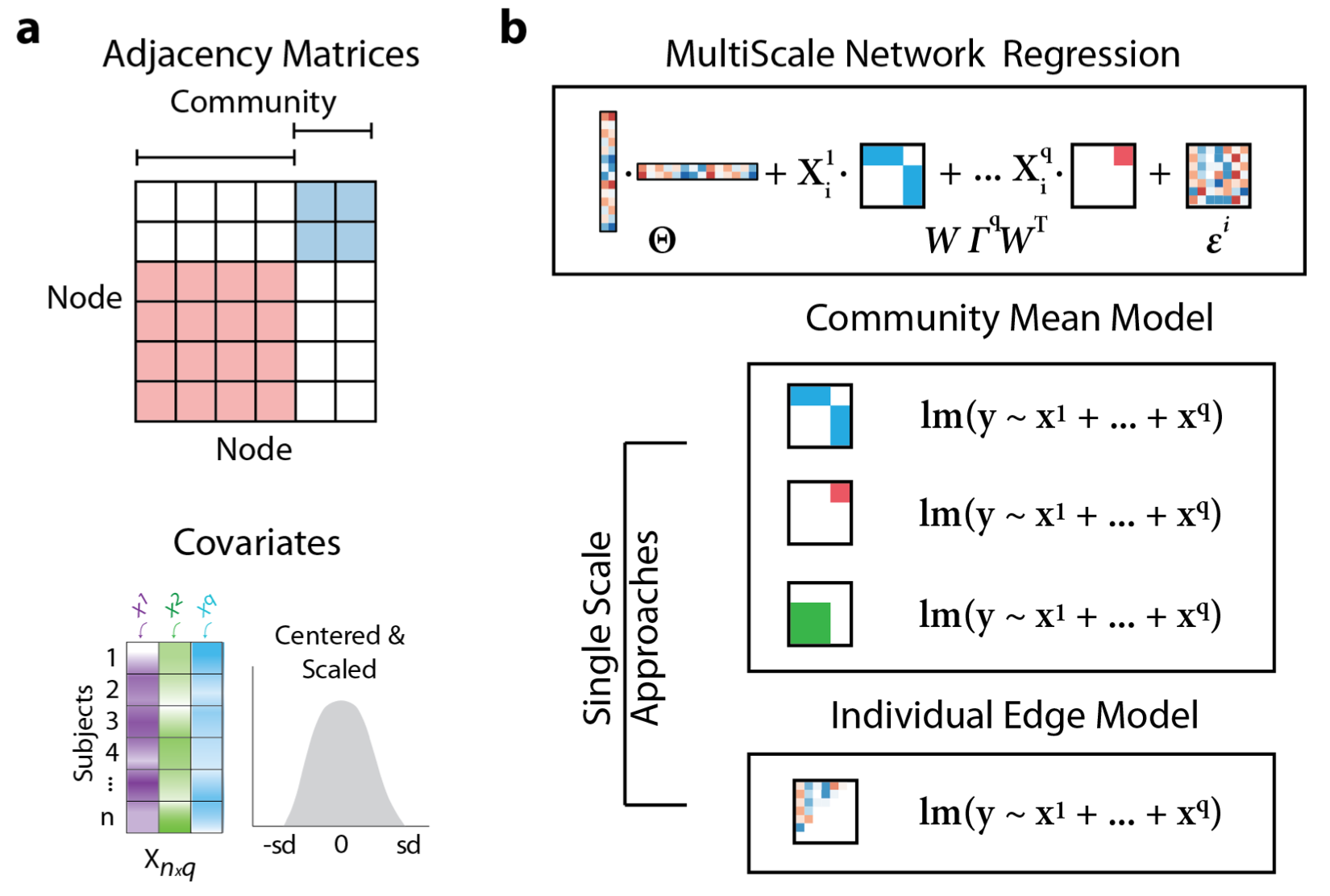


**Supplementary Figure 3 | Benchmarking MSNR against common single-scale approaches.** **a**) On the PNC data, we considered prediction of out-of-sample connectivity matrices from age, sex, and in-scanner motion. Specifically, input network data were $n \times p \times p$ connectivity matrices of $n$ subjects with $p$ nodes sorted a priori into $K$ communities. Additionally, covariate data were a $n \times q$ matrix of $q$ measurements, with each column centered with zero mean and scaled by its standard deviation. **b**) Specifically, we compared MSNR to two common network analysis approaches that only consider information present on a single scale. Linear models were fit for each edge or community connectivity for the individual edge and community mean model, respectively.


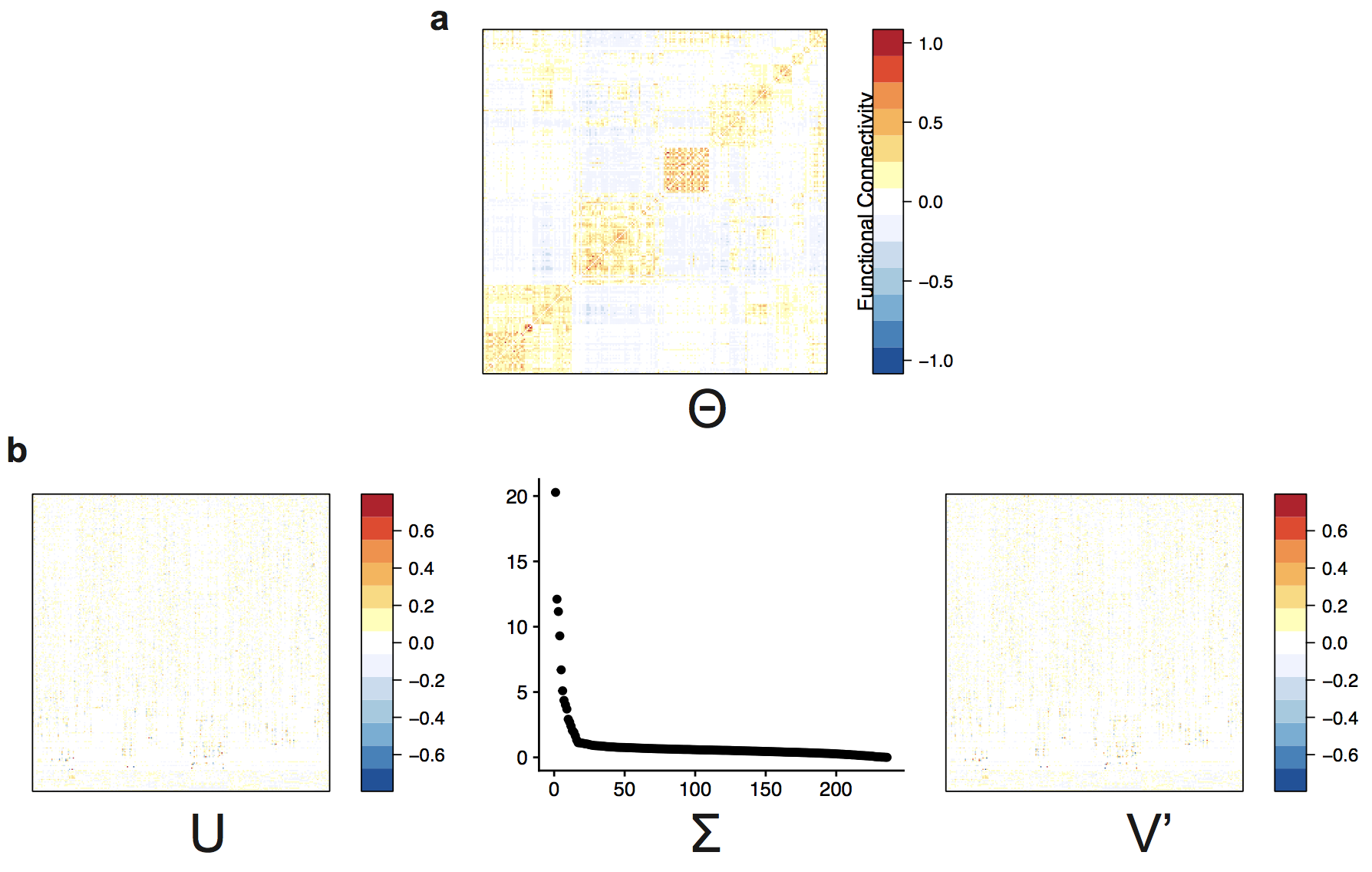


**Supplementary Figure 4 | Θ matrix in MSNR has a community structure and is low rank**. **a)** Θ matrix represents the mean connectivity matrix across all subjects in the model. It demonstrates a community structure as visualized here. **b)** Θ matrix is also low rank, as shown by singular value composition. This is consistent with the *Assumption 1* of MSNR.
